# Supplementary material for: Fecal Volatile Metabolomics Predict Gram-Negative Late-Onset Sepsis in Preterm Infants: A Nationwide Case-Control Study
Source: Microorganisms. 2023 Feb 24;11(3):572. doi: 10.3390/microorganisms11030572 (PMC10054547; doi:10.3390/microorganisms11030572)
Supplement: Supplementary file 1 [file microorganisms-11-00572-s001.zip › microorganisms-2233050-supplementary.pdf]

Supplementary Information

Supplementary Tables (S1–S4)

Table S1. Microbiology of blood cultures.

	**GC-IMS (n=121) N (%)**	**GC-TOF-MS (n=34) N (%)**
Gram-negative bacteria	31 (25.6)	15 (44.1)
Enterobacter aerogenes	2	2
Enterobacter cloacae	3	2
Escherichia coli	18	8
Klebsiella oxytoca	1	1
Klebsiella pneumoniae	4	2
Pseudomonas aeruginosa	1	0
Serratia marcescens	2	0
Gram-positive bacteria (excl. CoNS)	23 (19)	9 (26.5)
Streptococcus agalactiae	3	2
Staphylococcus aureus	16	7
Entercococcus faecalis	4	0
Coagulase negative *Staphylococci*	57 (47.1)	9 (26.5)
Staphylococcus capitis	8	3
Staphylococcus caprae	1	0
Staphylococcus epidermidis	41	4
Staphylococcus haemolyticus	5	2
Staphylococcus hominis	2	0
Multiple bacteria	8 (6.6)	1 (2.9)
Staphylococcus aureus + *Citrobacter freundii*	1	1
Staphylococcus epidermidis + *Staphylococcus capitis*	3	0
Staphylococcus epidermidis + *Bacillus cereus*	1	0
Staphylococcus epidermidis + *Staphylococcus hominis*	2	0
Escherichia coli + *Enterococcus faecalis*	1	0
Fungi	2 (1.7)	0 (0)
Candida albicans	2	0

Abbreviations: CoNS, coagulase negative *Staphylococci*; GC-TOF-MS, gas chromatography – time of flight/mass spectrometry; GC-IMS, gas chromatography – ion mobility spectrometry.

Table S2. Performance characteristics of GC-IMS as analyzed by random forest classification.

Subgroup	Number of Samples		*p*-value	AUC (± 95% CI)
	Case	Control		
**All categories of late-onset sepsis**				
**3 days before diagnosis (t_{-3})**	82	77	0.032	0.60 (0.53-0.67)
**2 days before diagnosis (t_{-2})**	79	100	0.0001	0.71 (0.64-0.77)
**1 day before diagnosis (t_{-1})**	89	101	0.019	0.60 (0.53-0.67)
**Combined time points $t_{-1-(-3)}$**	250	278	0.0001	0.68 (0.65-0.72)
1. Gram-negative late-onset sepsis				
**3 days before diagnosis (t_{-3})**	24	20	0.0001	**0.83 (0.72-0.93)***
**2 days before diagnosis (t_{-2})**	23	29	0.008	0.72 (0.58-0.84)
**1 day before diagnosis (t_{-1})**	25	26	0.147	0.62 (0.48-0.75)
**Combined time points $t_{-1-(-3)}$**	72	75	0.0001	**0.76 (0.70-0.83)***
a. E. coli late-onset sepsis				
**3 days before diagnosis (t_{-3})**	12	9	0.267	**0.77 (0.57-1.00)***
**2 days before diagnosis (t_{-2})**	12	14	0.001	**0.88 (0.74-1.00)***
**1 day before diagnosis (t_{-1})**	14	13	0.109	0.68 (0.49-0.86)
**Combined time points $t_{-1-(-3)}$**	38	36	0.056	0.63 (0.51-0.74)
2. Gram-positive late-onset sepsis (excluding coagulase-negative staphylococci)				
**3 days before diagnosis (t_{-3})**	17	21	0.872	0.52 (0.36-0.68)
**2 days before diagnosis (t_{-2})**	15	22	0.458	0.43 (0.27-0.59)
**1 day before diagnosis (t_{-1})**	21	20	0.297	0.60 (0.44-0.74)
**Combined time points $t_{-1-(-3)}$**	53	63	0.046	0.61 (0.52-0.70)
a. S. aureus late-onset sepsis				
**3 days before diagnosis (t_{-3})**	11	12	0.347	0.55 (0.29-0.81)
**2 days before diagnosis (t_{-2})**	8	13	0.788	0.60 (0.32-0.88)
**1 day before diagnosis (t_{-1})**	15	11	0.856	0.52 (0.30-0.73)
**Combined time points $t_{-1-(-3)}$**	34	35	0.045	0.62 (0.48-0.75)
3. Coagulase-negative staphylococci late-onset sepsis				
**3 days before diagnosis (t_{-3})**	39	36	0.895	0.49 (0.38-0.60)
**2 days before diagnosis (t_{-2})**	41	49	0.573	0.54 (0.43-0.63)
**1 day before diagnosis (t_{-1})**	43	54	0.017	0.64 (0.55-0.74)
**Combined time points $t_{-1-(-3)}$**	123	139	0.0001	0.68 (0.62-0.73)

Number of samples give an indication on the group sizes of the comparisons. *AUC ≥0.75. Abbreviations: LOS, late-onset sepsis; ±95% CI, 95% confidence interval; GC-IMS, Gas-chromatography – ion mobility spectrometry.

Table S3. Performance characteristics of GC-TOF-MS as analyzed by sparse logistic regression classification.

Subgroup	Number of samples: Case	Number of samples: Control	*p*-value	AUC (± 95% CI)
**All categories of late-onset sepsis**				
**3 days before diagnosis (t_{-3})**	26	27	0,69	0,55 (0,41-0,69)
**2 days before diagnosis (t_{-2})**	26	25	0,77	0,63 (0,49-0,77)
**1 day before diagnosis (t_{-1})**	27	27	0,76	0,64 (0,51-0,76)
**Combined time points $t_{-1-(-3)}$**	79	79	0,76	0,69 (0,62-0,76)
1. Gram-negative late-onset sepsis				
**3 days before diagnosis (t_{-3})**	11	13	0,89	0,73 (0,55-0,89)
**2 days before diagnosis (t_{-2})**	16	10	0,86	0,69 (0,48-0,86)
**1 day before diagnosis (t_{-1})**	11	12	0,72	0,52 (0,29-0,72)
**Combined time points $t_{-1-(-3)}$**	38	35	0,85	**0,77 (0,68-0,85)***
2. Gram-positive late-onset sepsis (excluding coagulase-negative staphylococci)				
**3 days before diagnosis (t_{-3})**	6	9	0,77	0,48 (0,20-0,77)
**2 days before diagnosis (t_{-2})**	4	7	1,00	0,64 (0,29-1.00)
**1 day before diagnosis (t_{-1})**	10	7	0,99	**0,75 (0,50-0,99)***
**Combined time points $t_{-1-(-3)}$**	20	23	0,86	0,74 (0,6-0,86)
3. Coagulase-negative staphylococci late-onset sepsis				
**3 days before diagnosis (t_{-3})**	9	5	0,89	0,52 (0,17-0,89)
**2 days before diagnosis (t_{-2})**	9	7	0,58	0,32 (0,08-0,58)
**1 day before diagnosis (t_{-1})**	7	8	0,84	0,55 (0,27-0,84)
**Combined time points $t_{-1-(-3)}$**	23	22	0,81	0,68 (0,54-0,81)

Number of samples give an indication on the group sizes of the comparisons. *AUC ≥0.75. Abbreviations: LOS, late-onset sepsis; ±95% CI, 95% confidence interval; GC-TOF-MS, Gas-chromatography – time of flight – mass spectrometry.

Table S4. Identified metabolites by GC-TOF-MS.

Metabolite	CAS-number	Chemical class	Detected in neonatal feces	Common in adult feces	Previous associations with disease
1-Propene, 2-methyl-	115-11-1	Hydrocarbons	X [60]		↑ UC remission [54] (in exhaled breath)
(2-Aziridinylethyl)amine	4025-37-0	Nitrogen containing			Associated with active UC [57]
Acetone	67-64-1	Ketones	X [60]	X [35,49,50,57, 60]	
Cyclopentane	287-92-3	Cycloalkanes			↓ UC remission [49]<br>↑ occurrence in CD infection [35]
Dimethyl ether	115-10-6	Ethers		X [50,51]	
Furan, 3-methyl-	930-27-8	Furans		X [35]	↓ in UC vs CrD and healthy patients [49]<br>↓ occurrence in UC and CD & CJ infections [35]
Isopropyl Alcohol	67-63-0	Alcohols	X [60]	X [35]	Associated with active IBD [52,57]<br>↑ in colorectal cancer [55]
Methylene chloride	75-09-2	Halomethanes		X [51]	↓ occurrence in UC [49]<br>↓ occurrence in UC and CD & CJ infections [35]
Propanal, 2-methyl-	78-84-2	Aldehydes	X [60]	X [35]	Associated with CrD [49]
Ethyl Acetate	141-78-6	Esters	X [60]		↑ in NAFLD [53]
Heptanal	111-71-7	Aldehydes	X [60]	X [35,57]	Associated with active CrD [52,57]<br>↑ occurrence in CJ infections [35]
Acetic acid, 2-(methylaminoethyl) ester	n.a.	Esters			
Propanoic acid, 2-hydroxy-, ethyl ester	97-64-3	Esters			
Propene	115-07-1	Alkenes			
2,3-Butanedione	431-03-8	Ketones	X [60]	X [35,53,57]	
Acetic acid	64-19-7	Acids	X [60,61]	X [57]	↑ in control vs LOS infants [61]<br>↓ active UC [56] (in exhaled breath)
Benzene	71-43-2	Hydrocarbons			↓ UC remission [49]<br>↑ occurrence UC [35]
Butanal, 3-methyl-	590-86-3	Aldehydes	X [60]	X [35,52,53]	
Butanoic acid, 3-methyl-	503-74-2	Fatty acids		X [35,57]	
Pentane, 2,2,4,4-tetramethyl-	1070-87-7	Alkanes			
Furan, 2-ethyl-	3208-16-0	Furans	X [60]		

The discriminatory metabolites are presented with their CAS-number and chemical class. This table shows whether the discriminatory metabolite has previously been detected in the feces of premature infants or adults. In addition, the relation of the metabolite to other diseases is depicted. X: The VOC is previously detected in neonatal feces or considered as common in adult feces. Abbreviations: GC-TOF-MS, gas chromatography – time of flight/mass spectrometry; UC, ulcerative colitis; CD, *Clostridium difficile*; CrD, Crohn's disease; CJ, *Campylobacter jejuni*; LOS, late-onset

sepsis; NAFLD, non-alcoholic fatty liver disease. n.a., not available. References are found in main text.

Supplementary Figures

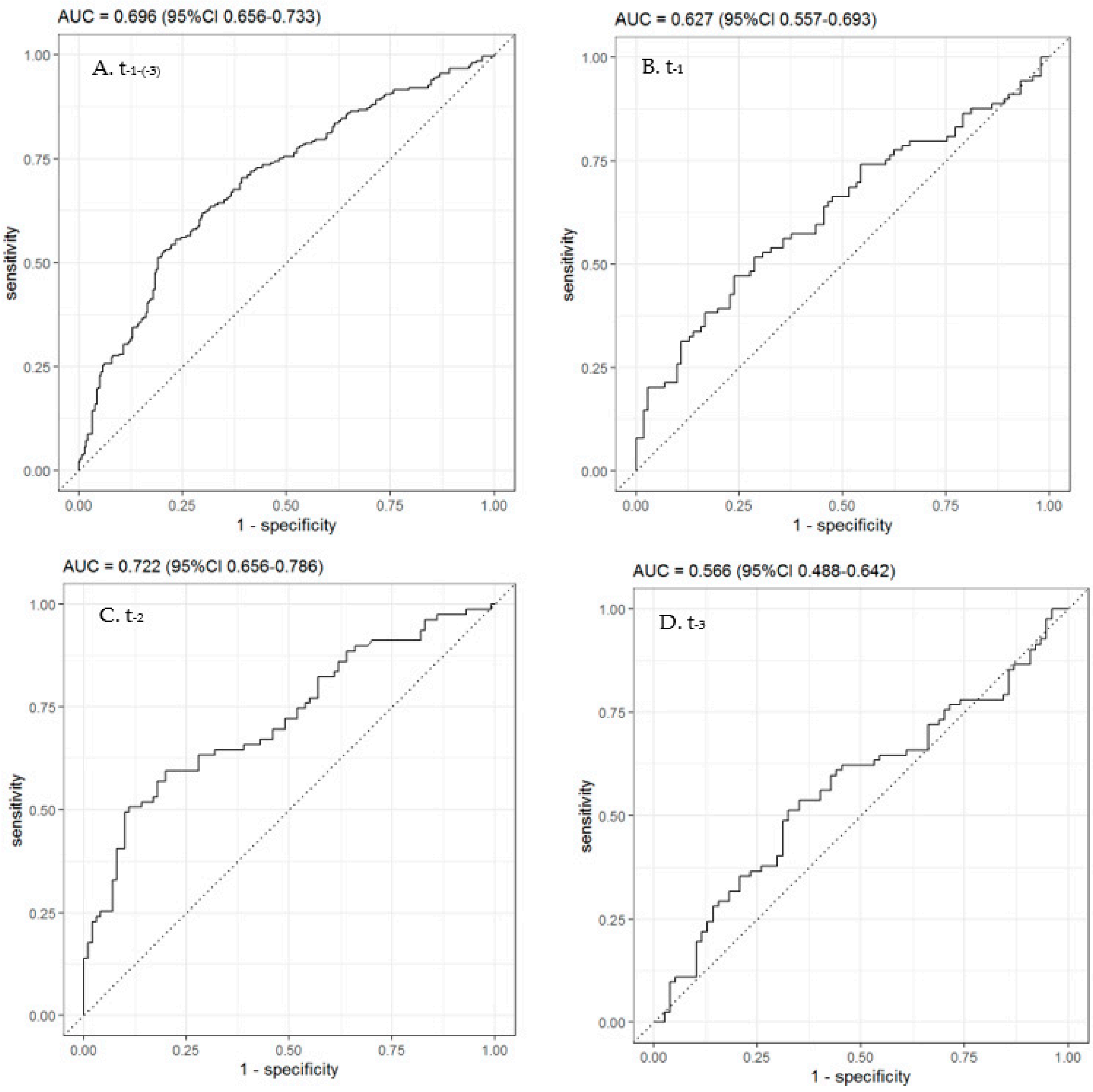

Figure S1. ROC curves for all LOS infants by GC-IMS. In this figure, the ROC curves with their corresponding AUC and 95% CI, are presented for all LOS infants as generated by sparse logistic regression algorithm. (A) All time points combined ($t_{-1-(-3)}$). B) 1 day prior to sepsis (t_{-1}). C) 2 days prior to sepsis (t_{-2}). D) 3 days prior to sepsis (t_{-3}). Abbreviations: LOS, late-onset sepsis; ROC, receiver operating characteristic; AUC, area under curve; 95% confidential intervals, 95% CI; GC-IMS, Gas-chromatography – ion mobility spectrometry.

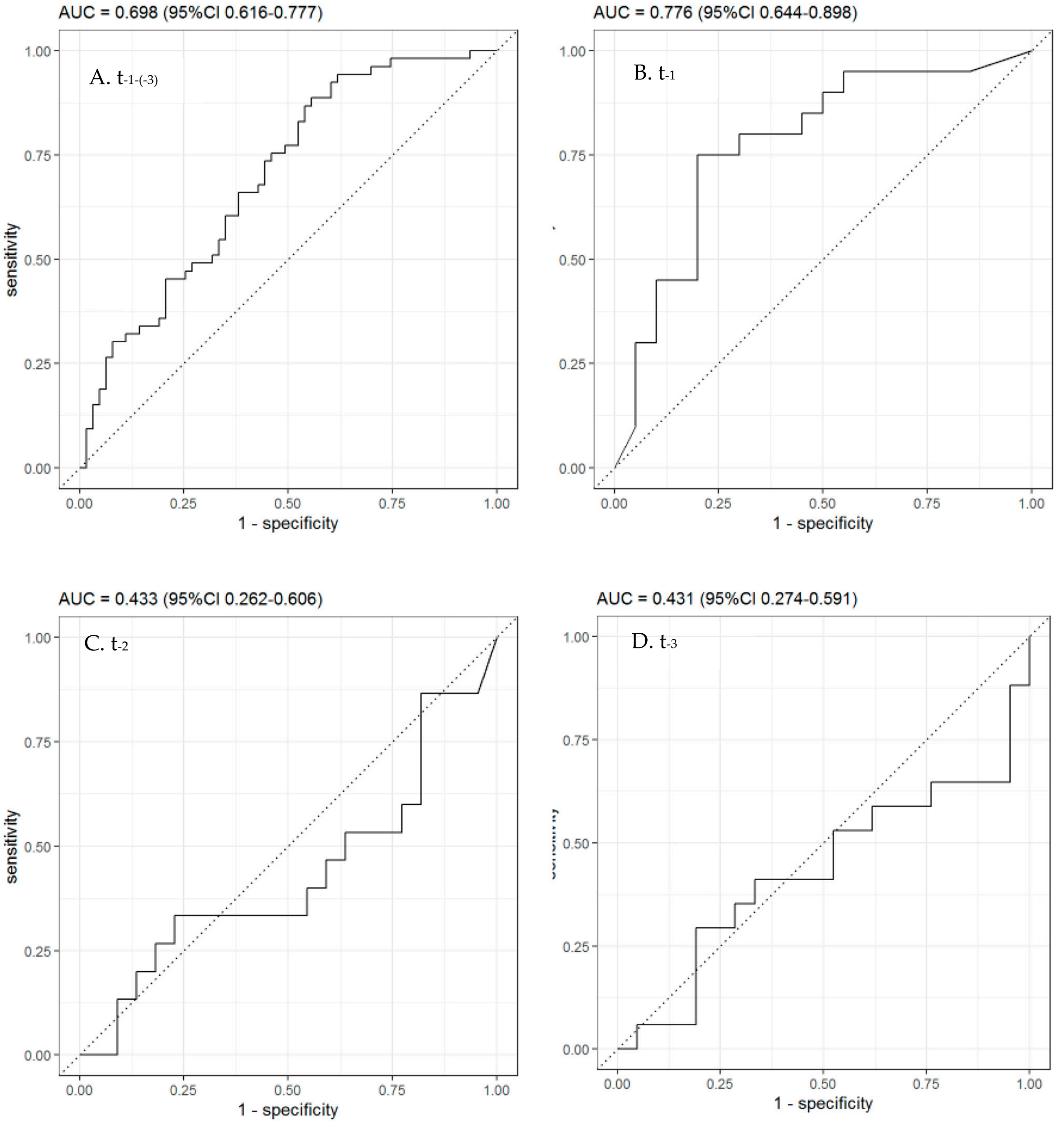

Figure S2. ROC curves for gram-positive LOS infants by GC-IMS. In this figure, the ROC curves with their corresponding AUC and 95% CI, are presented for gram-positive LOS infants as generated by sparse logistic regression algorithm. A) All time points combined ($t_{-1-(-3)}$). B) 1 day prior to sepsis (t_{-1}). C) 2 days prior to sepsis (t_{-2}). D) 3 days prior to sepsis (t_{-3}). Abbreviations: LOS, late-onset sepsis; ROC, receiver operating characteristic; AUC, area under curve; 95% confidential intervals, 95% CI; GC-IMS, Gas-chromatography – ion mobility spectrometry.

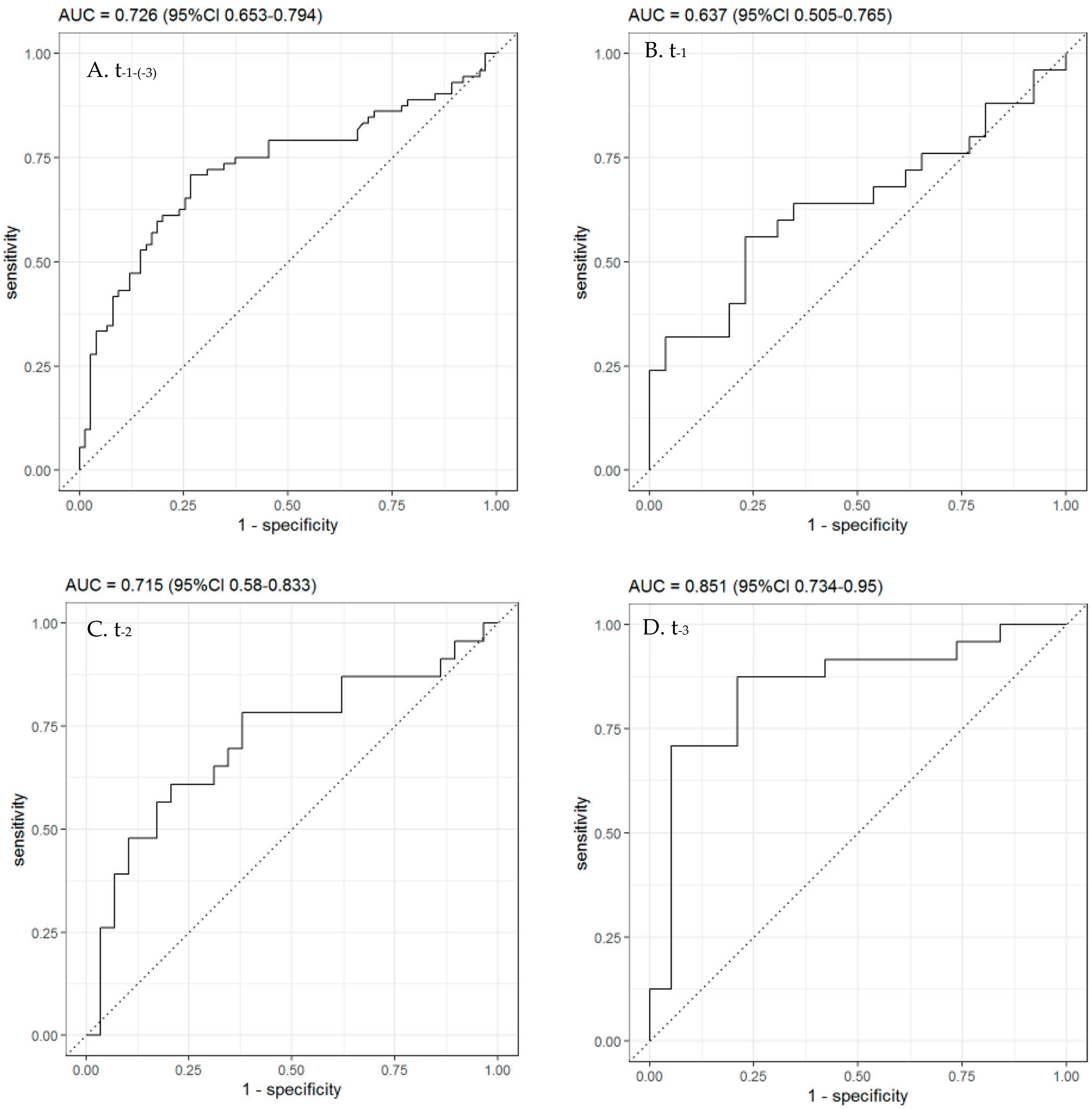

Figure S3. ROC curves for gram-negative LOS infants by GC-IMS. In this figure, the ROC curves with their corresponding AUC and 95% CI, are presented for gram-negative LOS infants as generated by sparse logistic regression algorithm. A) All time points combined ($t_{-1-(-3)}$). B) 1 day prior to sepsis (t_{-1}). C) 2 days prior to sepsis (t_{-2}). D) 3 days prior to sepsis (t_{-3}). Abbreviations: LOS, late-onset sepsis; ROC, receiver operating characteristic; AUC, area under curve; 95% confidential intervals, 95% CI; GC-IMS, Gas-chromatography – ion mobility spectrometry.

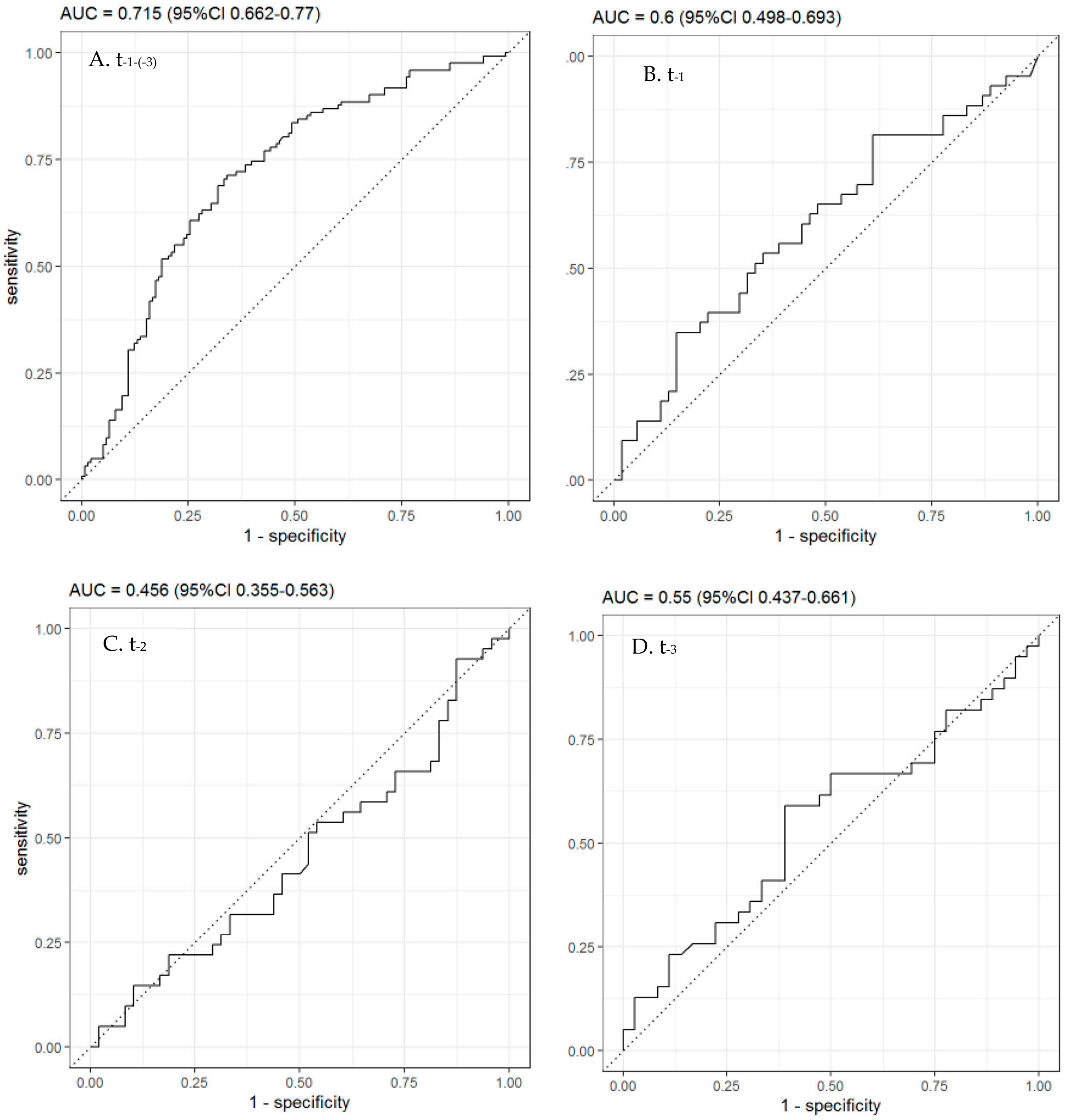

Figure S4. ROC curves for coagulase negative *staphylococcus* LOS infants by GC-IMS. In this figure, the ROC curves with their corresponding AUC and 95% CI, are presented for coagulase negative *staphylococcus* LOS infants as generated by sparse logistic regression algorithm. A) All time points combined ($t_{-1-(-3)}$). B) 1 day prior to sepsis (t_{-1}). C) 2 days prior to sepsis (t_{-2}). D) 3 days prior to sepsis (t_{-3}). Abbreviations: LOS, late-onset sepsis; ROC, receiver operating characteristic; AUC, area under curve; 95% confidential intervals, 95% CI; GC-IMS, Gas-chromatography – ion mobility spectrometry.

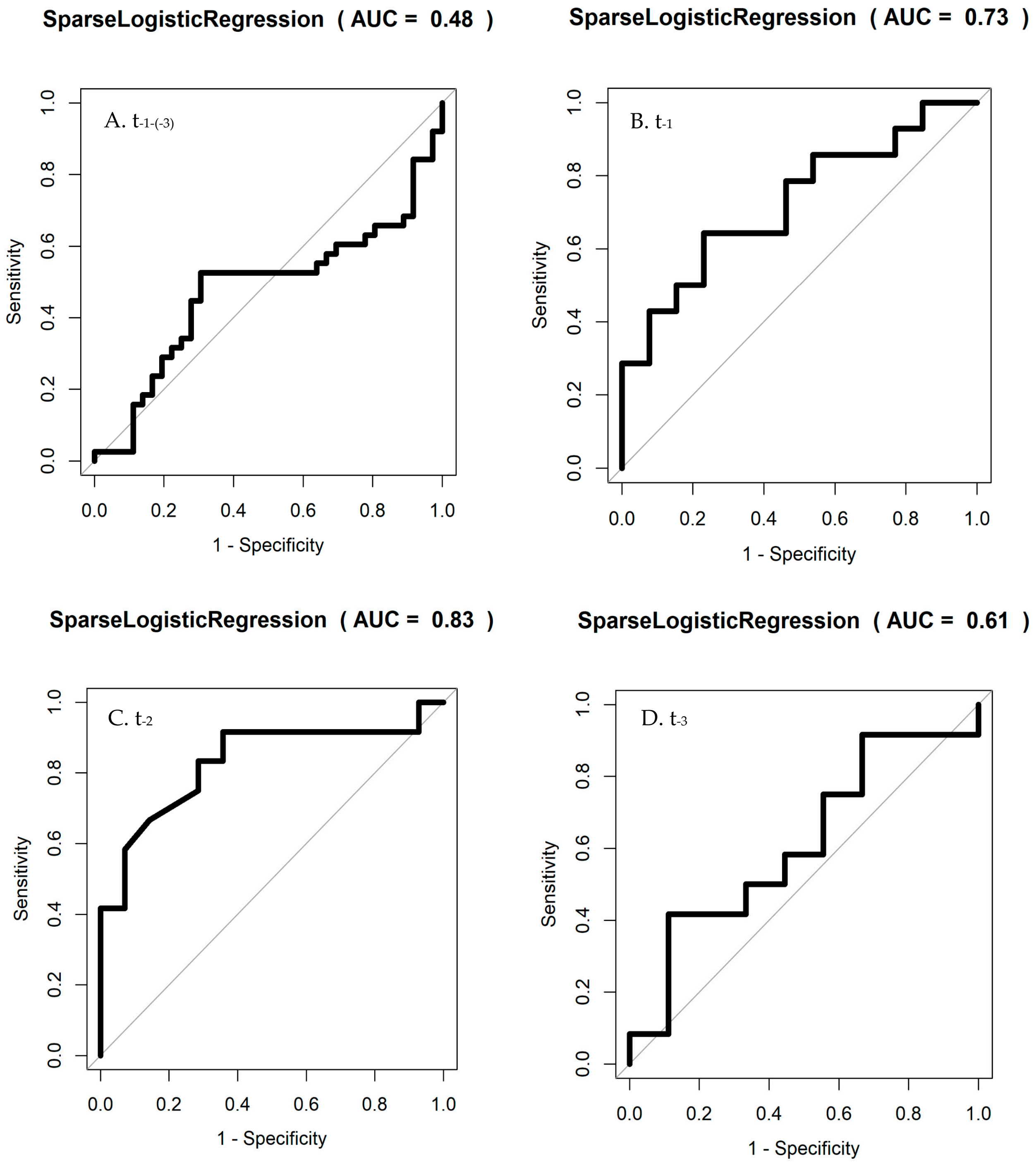

Figure S5. ROC curves for *E. coli* LOS infants by GC-IMS. In this figure, the ROC curves with their corresponding AUC and 95% CI, are presented for *E. coli* LOS infants as generated by sparse logistic regression algorithm. A) All time points combined ($t_{-1-(-3)}$). B) 1 day prior to sepsis (t_{-1}). C) 2 days prior to sepsis (t_{-2}). D) 3 days prior to sepsis (t_{-3}). Abbreviations: LOS, late-onset sepsis; ROC, receiver operating characteristic; AUC, area under curve; 95% confidential intervals, 95% CI; GC-IMS, Gas-chromatography – ion mobility spectrometry.

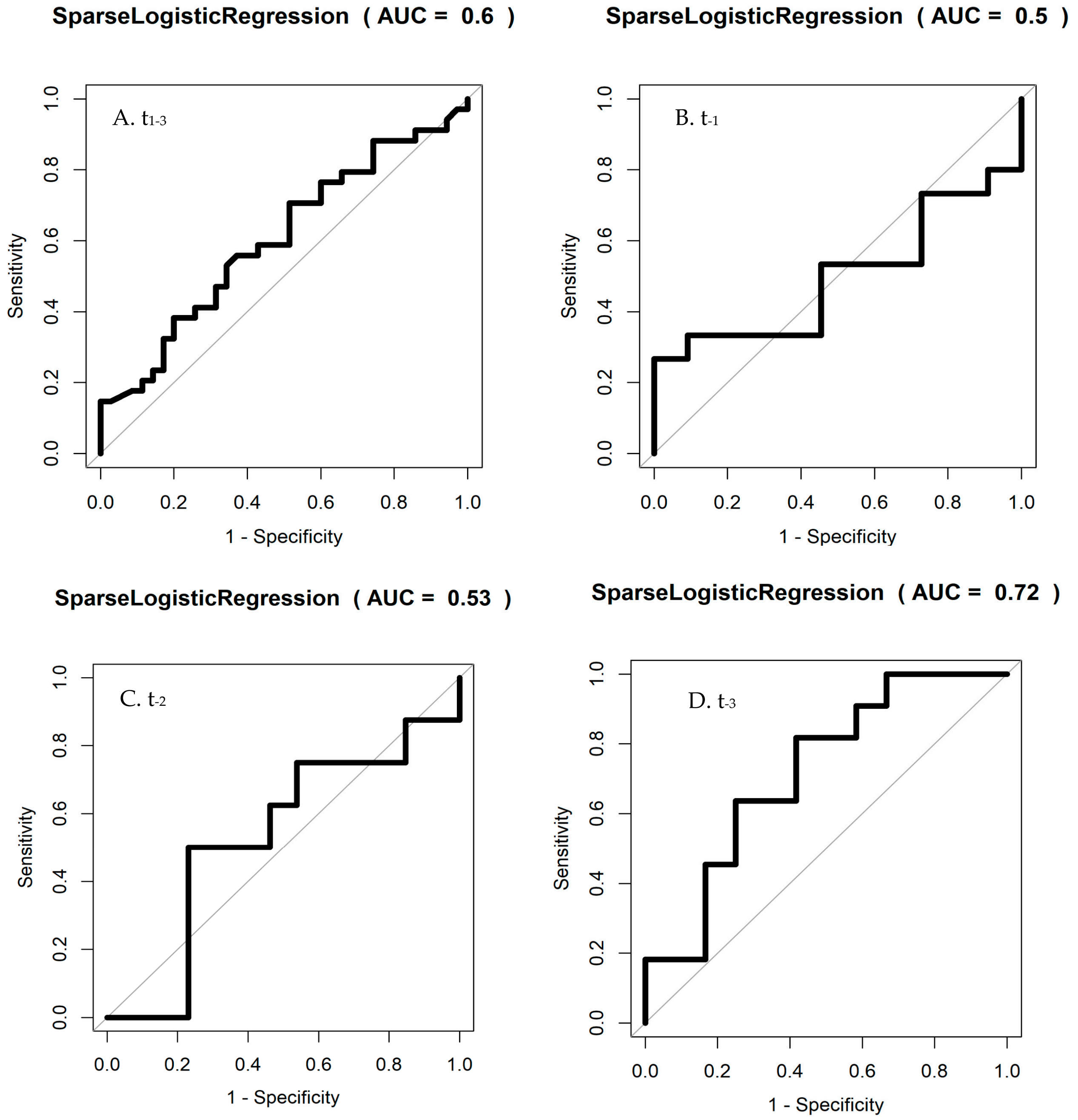

Figure S6. ROC curves for *S. aureus* LOS infants by GC-IMS. In this figure, the ROC curves with their corresponding AUC and 95% CI, are presented for *S. aureus* LOS infants as generated by sparse logistic regression algorithm. A) All time points combined ($t_{-1-(-3)}$). B) 1 day prior to sepsis (t_{-1}). C) 2 days prior to sepsis (t_{-2}). D) 3 days prior to sepsis (t_{-3}). Abbreviations: LOS, late-onset sepsis; ROC, receiver operating characteristic; AUC, area under curve; 95% confidential intervals, 95% CI; GC-IMS, Gas-chromatography – ion mobility spectrometry.

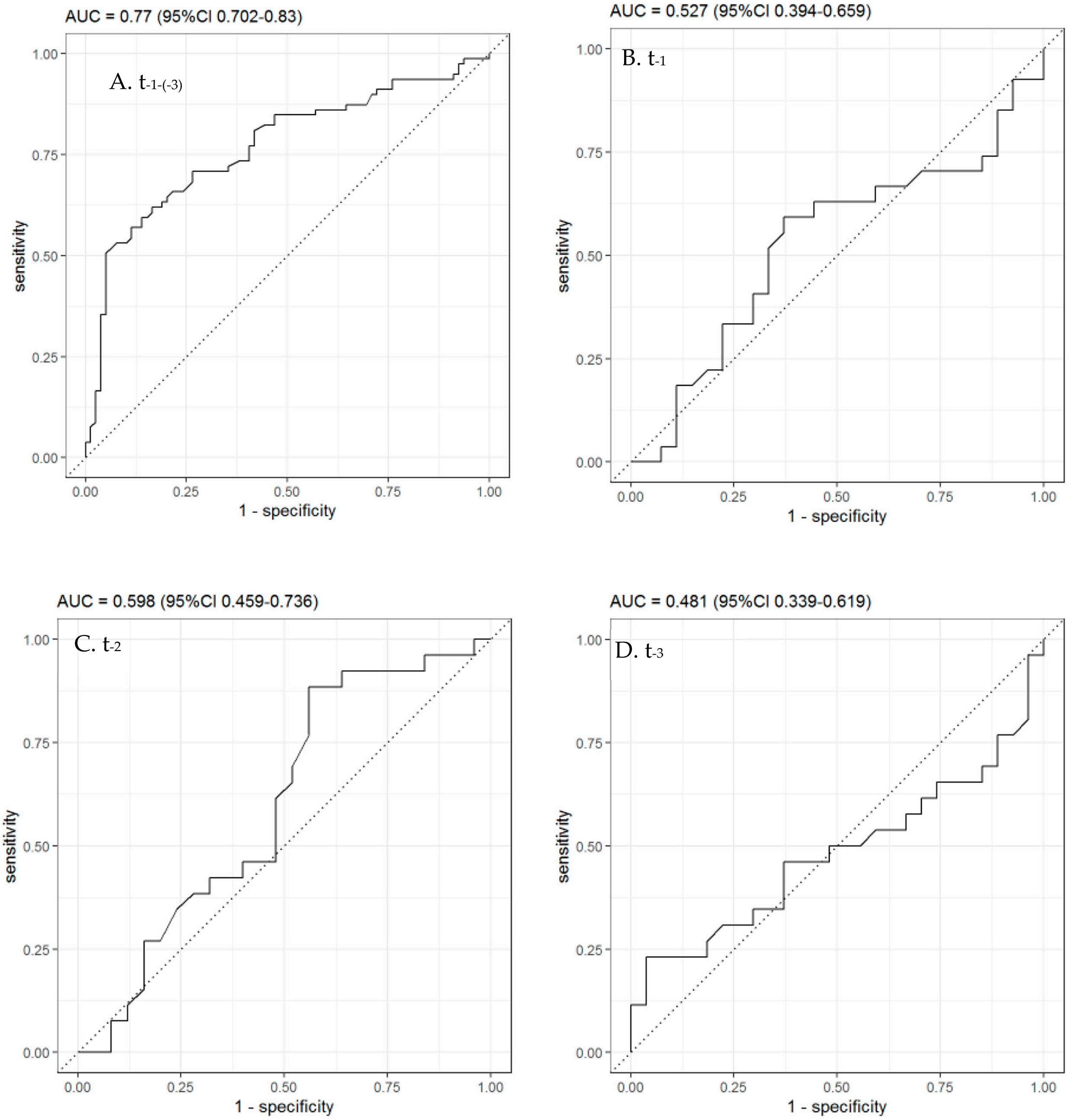

Figure S7. ROC curves for all LOS infants by GC-TOF-MS. In this figure, the ROC curves generated by the random forest classification algorithm are presented with corresponding area under the curves (AUC) and 95% confidential intervals (95% CI). A) All time points combined ($t_{-1-(-3)}$). B) 1 day prior to sepsis (t_{-1}). C) 2 days prior to sepsis (t_{-2}). D) 3 days prior to sepsis (t_{-3}). Abbreviations: LOS, late-onset sepsis; ROC, receiver operating characteristic; GC-TOF-MS, Gas-chromatography – time of flight – mass spectrometry.

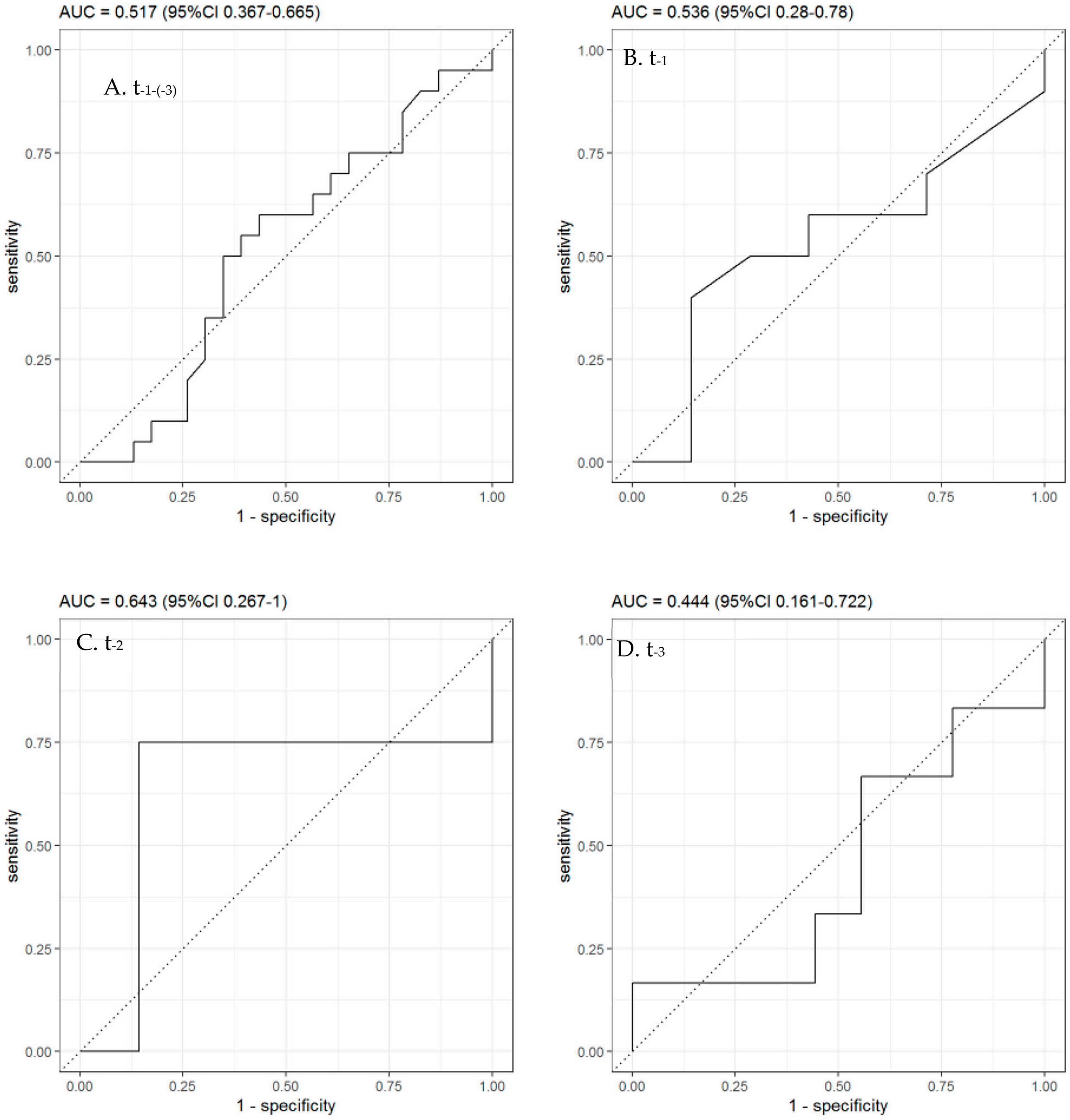

Figure S8. ROC curves for gram-positive LOS infants by GC-TOF-MS. In this figure, the ROC curves generated by the random forest classification algorithm are presented with corresponding area under the curves (AUC) and 95% confidential intervals (95% CI). A) All time points combined ($t_{-1-(-3)}$). B) 1 day prior to sepsis (t_{-1}). C) 2 days prior to sepsis (t_{-2}). D) 3 days prior to sepsis (t_{-3}). Abbreviations: LOS, late-onset sepsis; ROC, receiver operating characteristic; GC-TOF-MS, Gas-chromatography – time of flight – mass spectrometry.

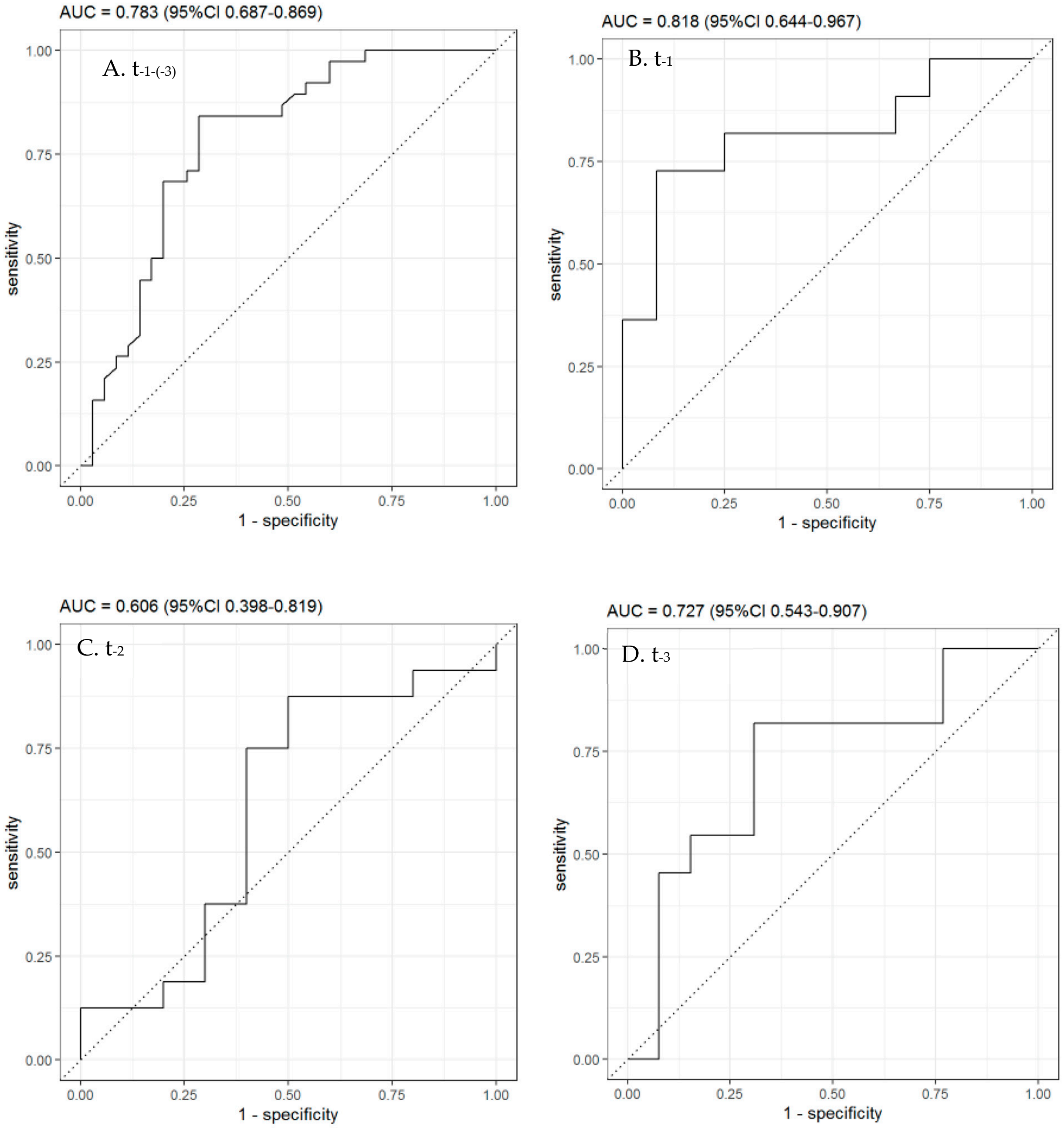

Figure S9. ROC curves for gram-negative LOS infants by GC-TOF-MS. In this figure, the ROC curves generated by the random forest classification algorithm are presented with corresponding area under the curves (AUC) and 95% confidential intervals (95% CI). A) All time points combined ($t_{-1-(-3)}$). B) 1 day prior to sepsis (t_{-1}). C) 2 days prior to sepsis (t_{-2}). D) 3 days prior to sepsis (t_{-3}). Abbreviations: LOS, late-onset sepsis; ROC, receiver operating characteristic; GC-TOF-MS, Gas-chromatography – time of flight – mass spectrometry.

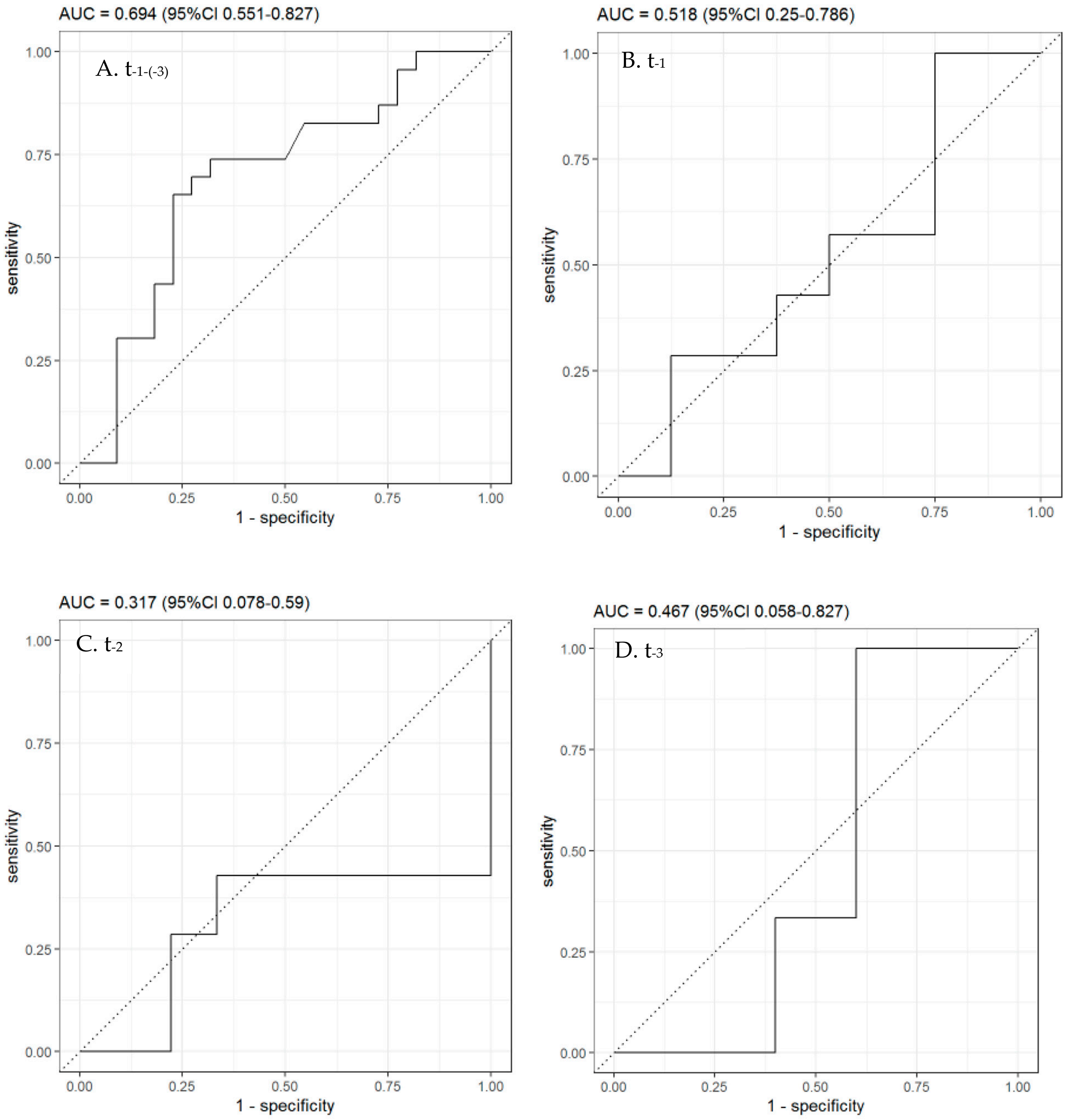

Figure S10. ROC curves for coagulase negative *staphylococcus* LOS infants by GC-TOF-MS. In this figure, the ROC curves generated by the random forest classification algorithm are presented with corresponding area under the curves (AUC) and 95% confidential intervals (95% CI). A) All time points combined ($t_{-1-(-3)}$). B) 1 day prior to sepsis (t_{-1}). C) 2 days prior to sepsis (t_{-2}). D) 3 days prior to sepsis (t_{-3}). Abbreviations: LOS, late-onset sepsis; ROC, receiver operating characteristic; GC-TOF-MS, Gas-chromatography – time of flight – mass spectrometry.
